# Supplementary figures and images for: Comparative genomics of the closely related fungal genera Cryptococcus and Kwoniella reveals karyotype dynamics and suggests evolutionary mechanisms of pathogenesis
Source: PLoS Biol. 2024 Jun 6;22(6):e3002682. doi: 10.1371/journal.pbio.3002682 (PMC11185503; doi:10.1371/journal.pbio.3002682)

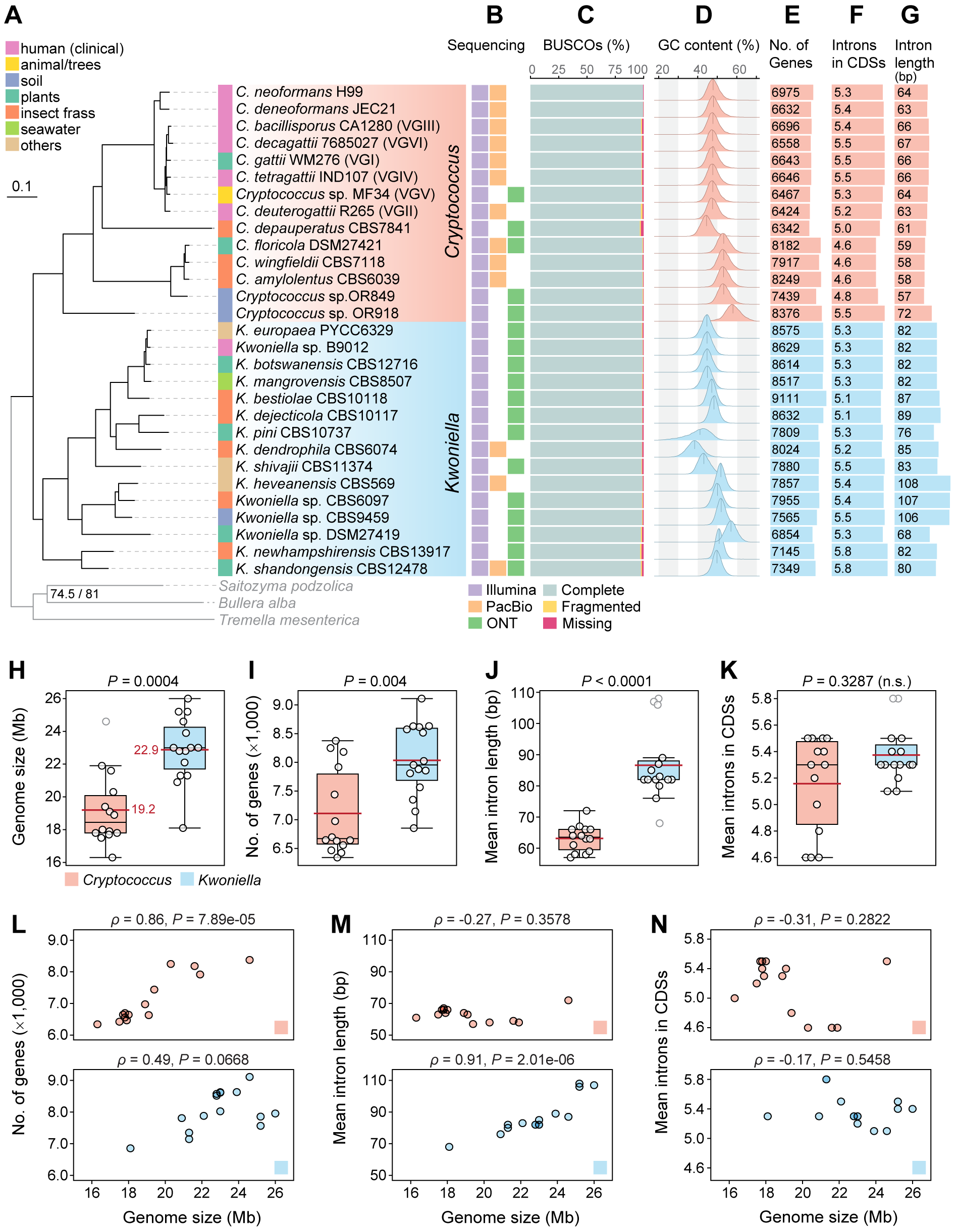

Supplement: S1 Fig — (A) Maximum likelihood phylogeny of Cryptococcus and Kwoniella inferred through a concatenation-based approach on a data matrix composed of protein alignments of 3,430 single-copy genes shared across all species and 3 outgroups (depicted in grey). Except where indicated, all branches are 100% supported (SH-aLRT and UFboot tests). Branch lengths are given in number of substitutions per site (scale bar). The isolation origin of each strain is indicated as given in the key. (B) Genome sequencing approach for each of the strains. (C) BUSCO completeness assessment of each genome gene set. (D) Frequency distribution of GC content across species, with mean GC values represented by vertical lines. (E) Number of genes, (F) mean number of introns within coding sequences (CDSs), and (G) their mean length (in base pairs, bp). Box plot comparisons of (H) genome sizes, (I) number of genes, (J) mean intron length, and (K) mean number of introns in CDSs, between Cryptococcus and Kwoniella. The red line, black line, boxes, and grey circles denote the mean value, median value, interquartile range, and outliers, respectively. P values obtained by Mann–Whitney U test; n.s., not significant. (L, M) Comparative analysis of gene count, intron length, and mean number of introns relative to total genome size. Each plot shows Spearman’s correlation coefficients (ρ) and associated P values indicating the strength and direction of these relationships. The data underlying this Figure can be found in S1 Appendix and at https://doi.org/10.5281/zenodo.11199354. (TIF) [file pbio.3002682.s001.tif]

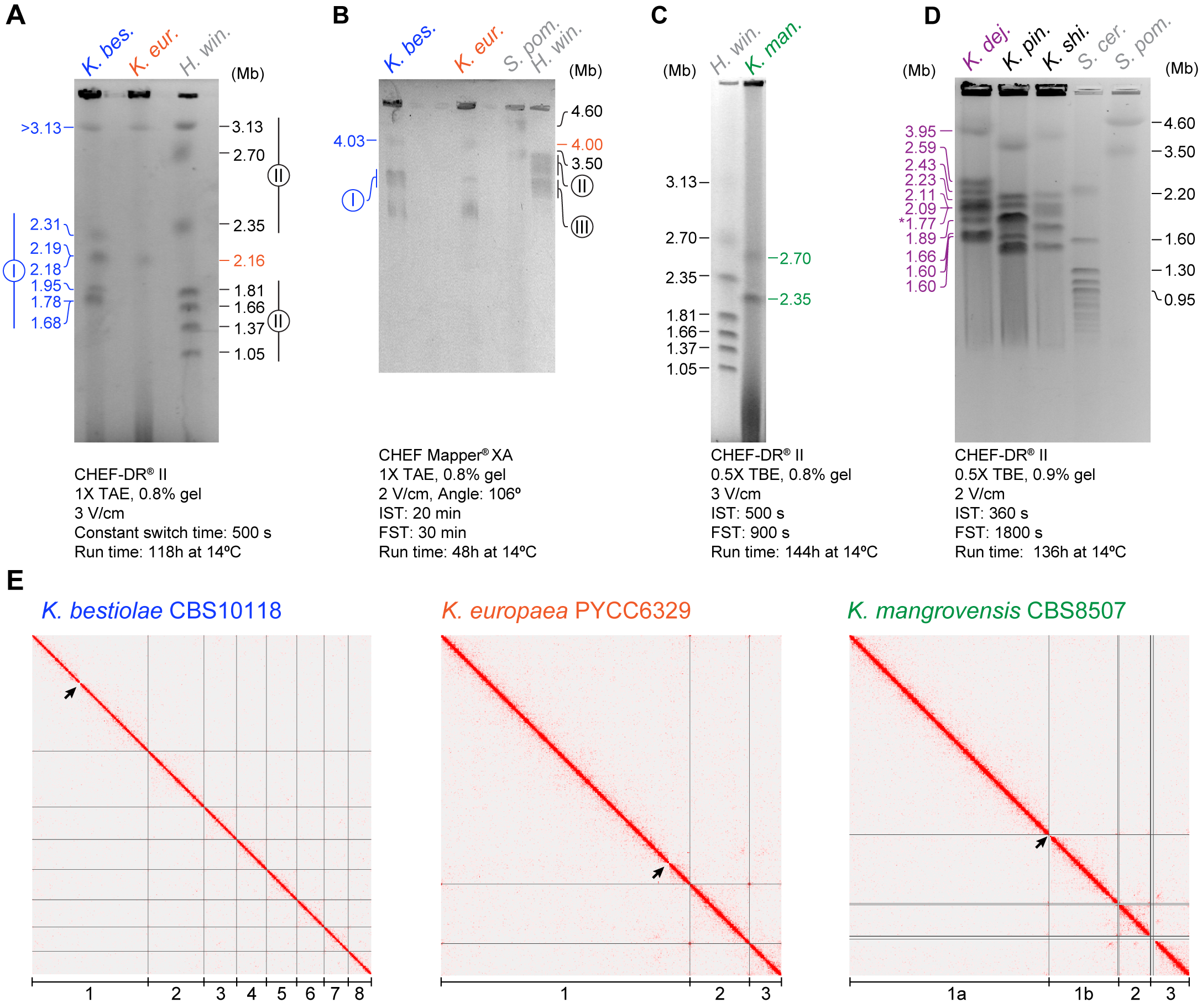

Supplement: S2 Fig — (A-D) Electrophoretic karyotypes of selected Kwoniella species with different number of chromosomes: K. bestiolae with 8 chrs (blue); K. europaea and K. mangrovensis with 3 chrs. each (orange and green, respectively); and K. dejecticola (purple), K. pini, and K. shivajii with 11 chrs. each. Saccharomyces cerevisiae, Schizosaccharomyces pombe, and Hansenula wingei chromosomes serve as markers, with their sizes in megabase pairs (Mb) shown in black. Color-coded numbers indicate contigs sizes in each respective assembly. Two running conditions were used for better separation of small and large chromosomes in K. bestiolae and K. europaea (panels A and B). The largest chromosomes in K. bestiolae (8.41 Mb), K. europaea (16.67 Mb), and K. mangrovensis (18.17 Mb) are too large to be resolved by this approach. The karyotypes of K. dejecticola, K. pini, and K. shivajii confirm 11 chrs. in each species, aligning with the contig number and length. The contig size harboring the rDNA array in K. dejecticola (indicated by an asterisk) is likely underestimated. (E) Hi-C contact matrix showing interaction frequencies between genomic regions, with pixel intensity indicating how often a pair of loci interact. Most of the links are nearby intrachromosomal, validating our assemblies. Interaction frequencies produced with Juicer Tools v1.7.6 are summarized along the genome. Chromosome numbers are given at the bottom of each plot. In K. mangrovensis, chr. 1 is broken at the rDNA array (black arrow in each plot) and shown as 2 contigs (1a and 1b). (TIF) [file pbio.3002682.s002.tif]

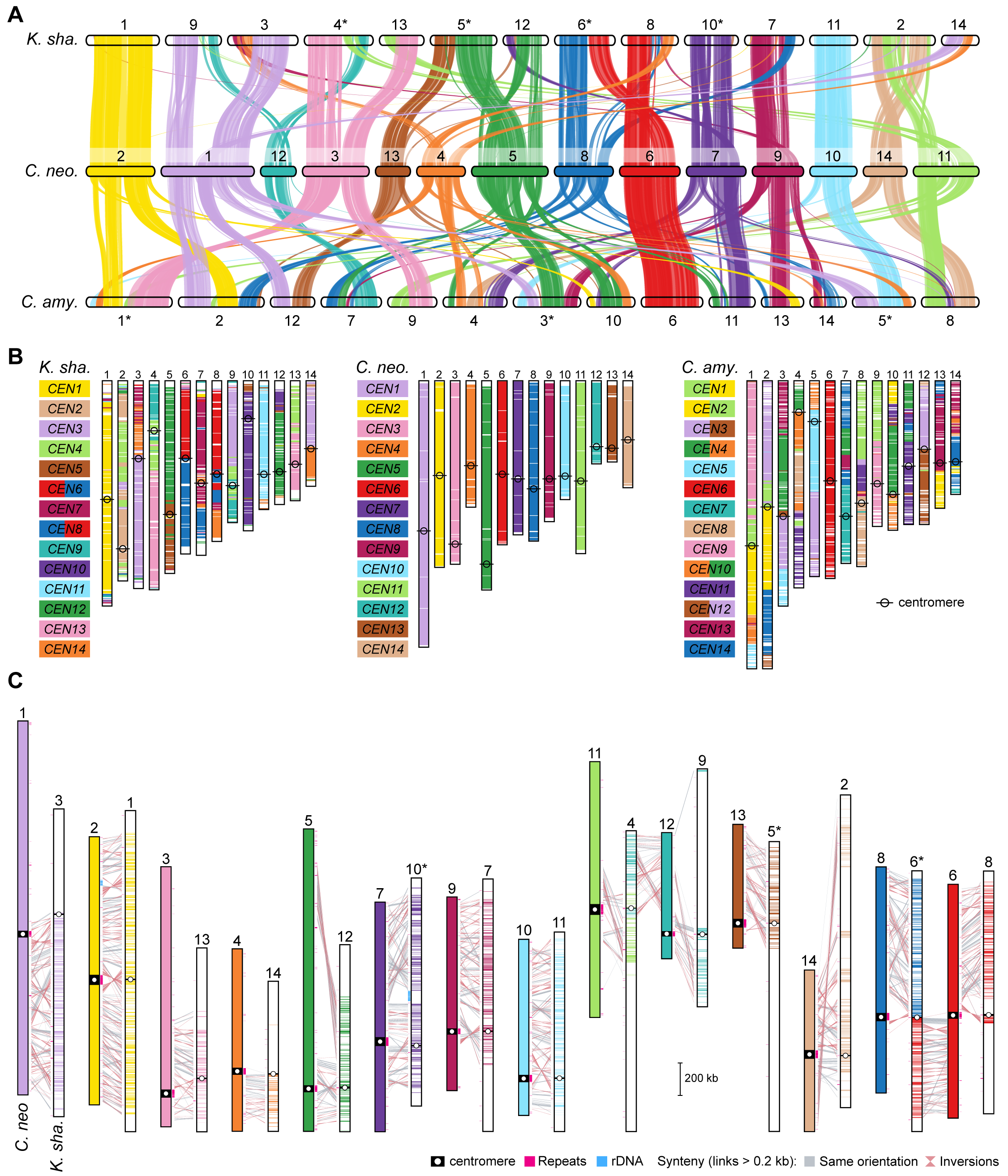

Supplement: S3 Fig — (A) Pairwise synteny relationships between C. neoformans, C. amylolentus, and K. shandongensis, all with 14 chromosomes. Links depict boundaries of syntenic gene blocks identified by MCScanX, with pairwise homologous relationships determined by SynChro. Chromosomes are color-coded based on C. neoformans and were reordered or inverted (marked with asterisks) from their original assembly orientations to maximize collinearity. (B) Superimposition of synteny blocks and centromere locations reveals 3 intercentromeric rearrangements between C. neoformans and C. amylolentus, as opposed to a single one between C. neoformans and K. shandongensis. (C) Synteny analysis based on BLASTN comparing chromosomal regions encompassing centromeres in K. shandongensis (predicted in silico) relative to previously determined centromeres of C. neoformans. Despite the numerous intrachromosomal rearrangements between these 2 species, centromere-flanking regions exhibit full (e.g., CnCEN2) or at least partial (e.g., CnCEN6/8) synteny. (TIF) [file pbio.3002682.s003.tif]

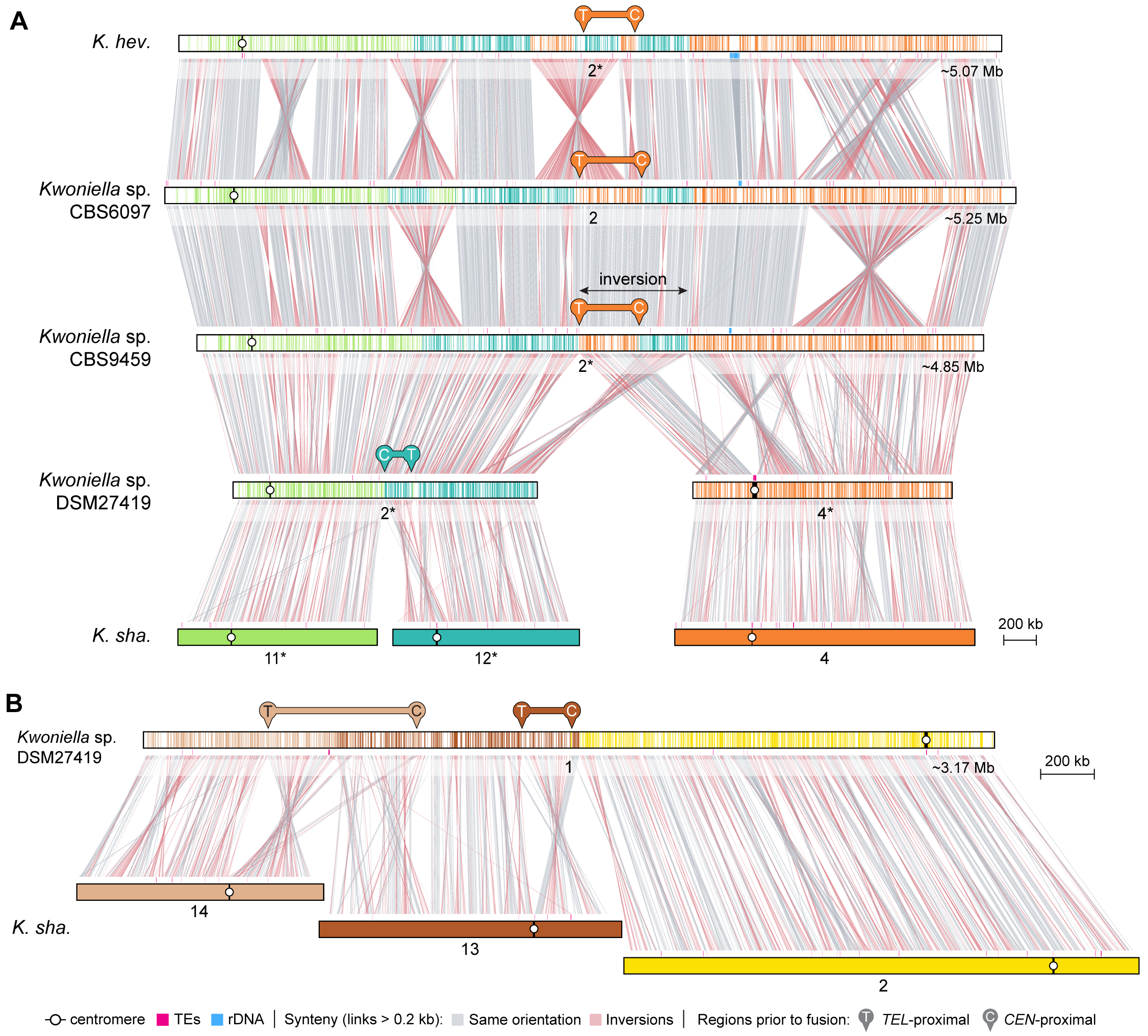

Supplement: S4 Fig — Synteny comparison showing that (A) chr. 2 of Kwoniella sp. DSM27419 resulted from the fusion of K. shandongensis chrs. 11 and 12. and that (B) chr. 1 of Kwoniella sp. DSM27419 emerged from the fusion of 3 chromosomes extant in K. shandongensis (chrs. 2, 13, and 14), followed by several intrachromosomal rearrangements. These 2 fusion events, inferred as the oldest within Kwoniella (event A in Fig 2), resulted in chromosome arrangements consistent across all species after the split from K. shandongensis/K. newhampshirensis (albeit with a few subsequent species-specific rearrangements). Note that the centromere-proximal regions of K. shandongensis chromosomes align at or near the fusion points on Kwoniella sp. DSM27419 fused chromosomes, while telomere-proximal regions are more internal, suggesting large inversions targeting centromeric regions accompanied each fusion event. In K. heveanensis, and sibling species Kwoniella sp. CBS6097 and Kwoniella sp. CBS9495, chr. 2 results from a subsequent fusion of K. shandongensis chr. 4 to the already fused chr. 11–12 (event D in Fig 2), followed by several intrachromosomal rearrangements. This event occurred in the common ancestor of these 3 species and the centromere- and telomere-proximal regions have been inverted back by a secondary inversion (double-sided black arrow) that occurred after the initial fusion. Chromosomes inverted from their original assembly orientations are marked with asterisks. (TIF) [file pbio.3002682.s004.tif]

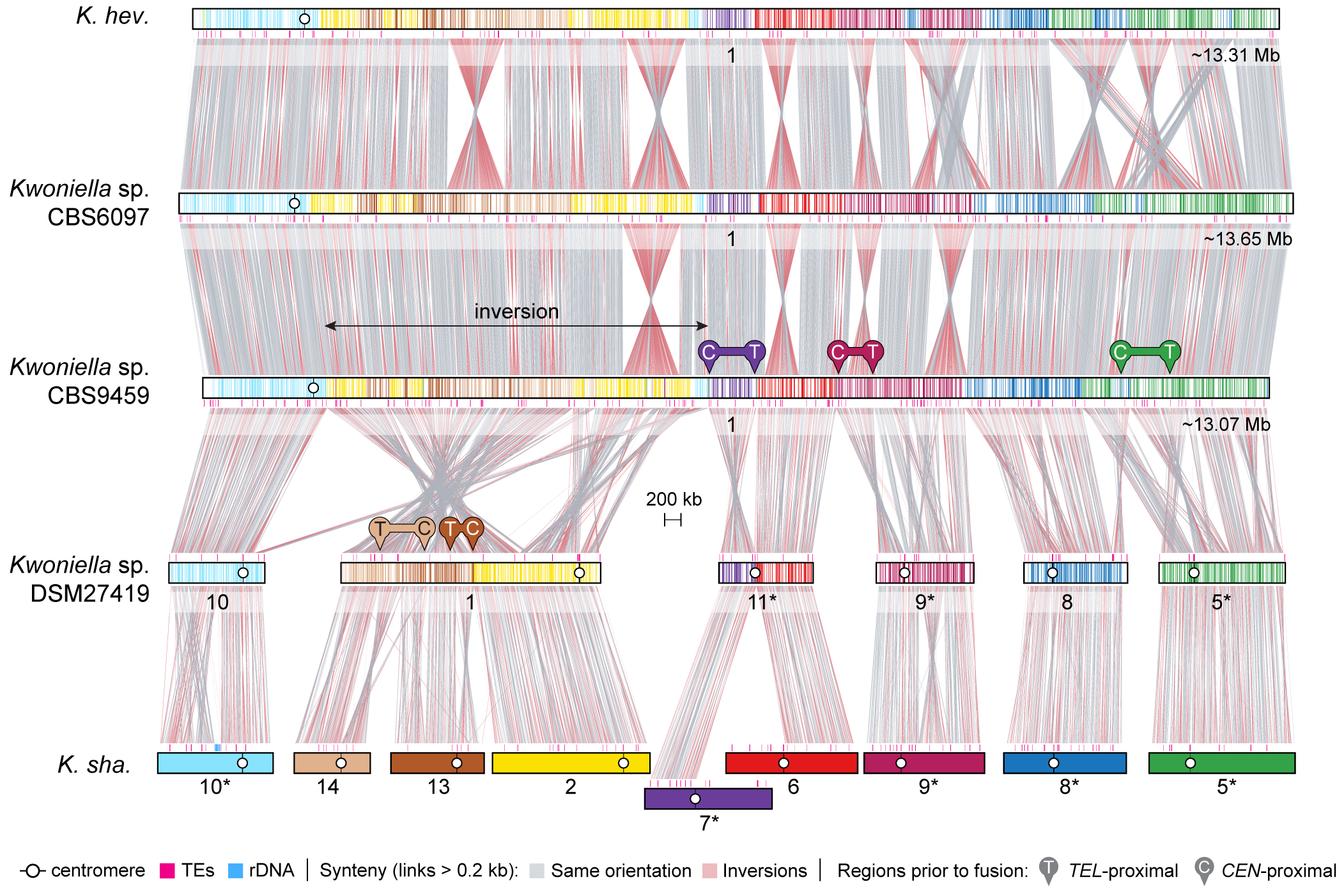

Supplement: S5 Fig — Synteny comparison showing that chr. 1 of K. heveanensis and sibling species Kwoniella sp. CBS6097 and Kwoniella sp. CBS9495 resulted from fusion of 6 chromosomes, followed by several intrachromosomal rearrangements. Three of the ancestral chromosomes had been fused prior to this event (chrs. 2-13-14). Note that most of centromere-proximal regions of K. shandongensis chromosomes are located at or near the fusion points on the giant chromosome, whereas the telomere-proximal regions are more internalized, suggesting that a large inversion targeting the centromeric region is associated with each fusion event. Chr. 11 of Kwoniella sp. DSM27419 resulted from reciprocal translocation between K. shandongensis chrs. 6 and 7 (event C in Fig 2). (TIF) [file pbio.3002682.s005.tif]

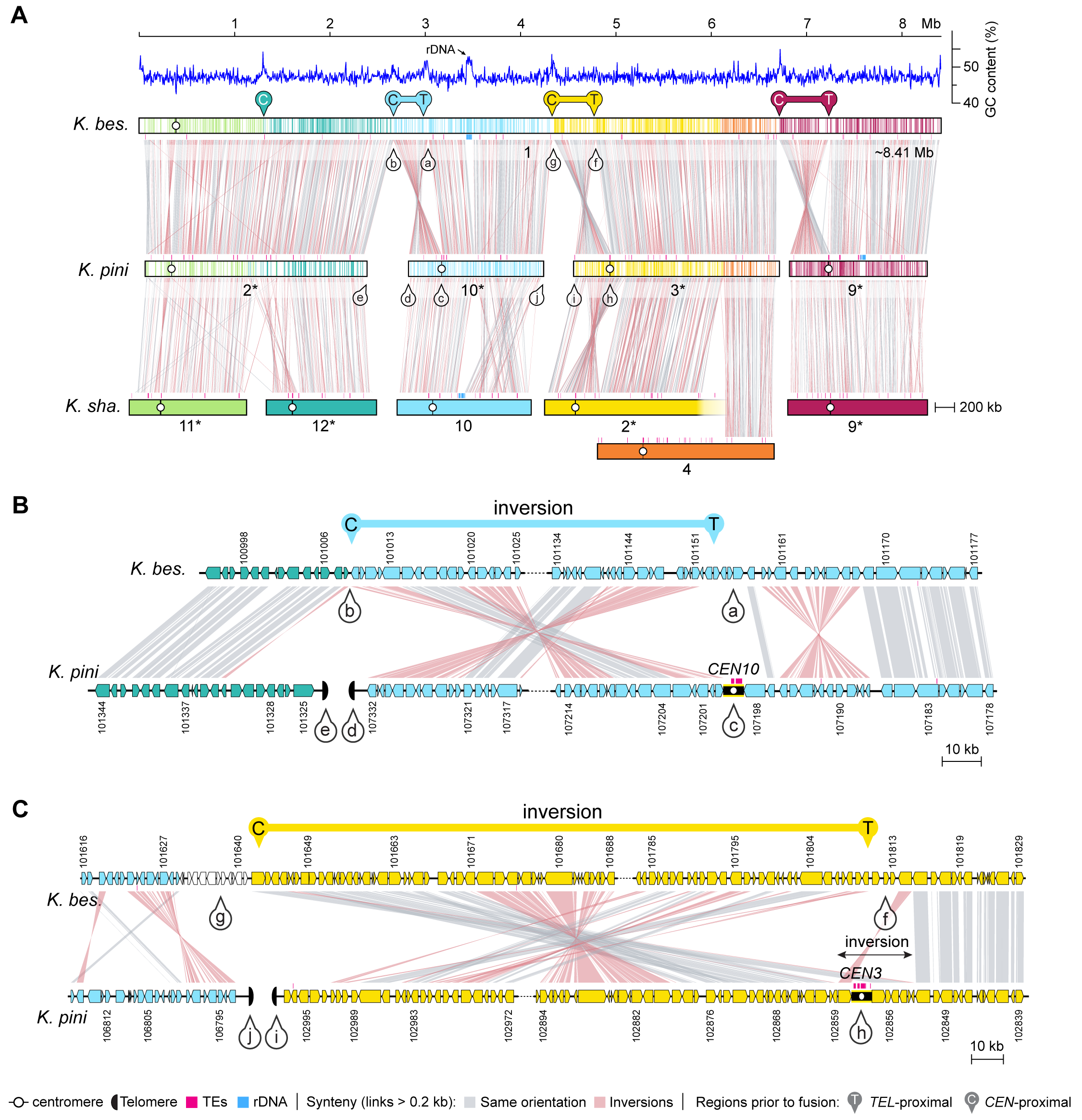

Supplement: S6 Fig — (A) Synteny comparison showing that K. bestiolae chr. 1 resulted from fusion of 4 chromosomes, extant in K. pini. Two of the ancestral chromosomes had been fused prior to this event (chrs. 11 and 12). K. pini chr. 3 resulted from a translocation between K. shandongensis chr. 4 and an ancestrally formed chromosome resulting from fusion of K. shandongensis chrs. 2, 13, and 14 (event B in Fig 2). (B, C) Zoomed-in synteny views of the regions marked in panel A (pins with lowercase letters from a–h). Note that most of centromere-proximal regions of K. pini chromosomes are located at or near the fusion points on the giant chromosome, whereas the telomere-proximal regions are more internalized, suggesting that a large inversion targeting the centromeric region is associated with each fusion event. A secondary inversion (double-sided black arrow) likely occurred in K. pini reversing the relative orientation of the centromere and a few flanking genes. Species-specific differences in gene content and relative orientation are expected as these species have diverged for a long time. (TIF) [file pbio.3002682.s006.tif]

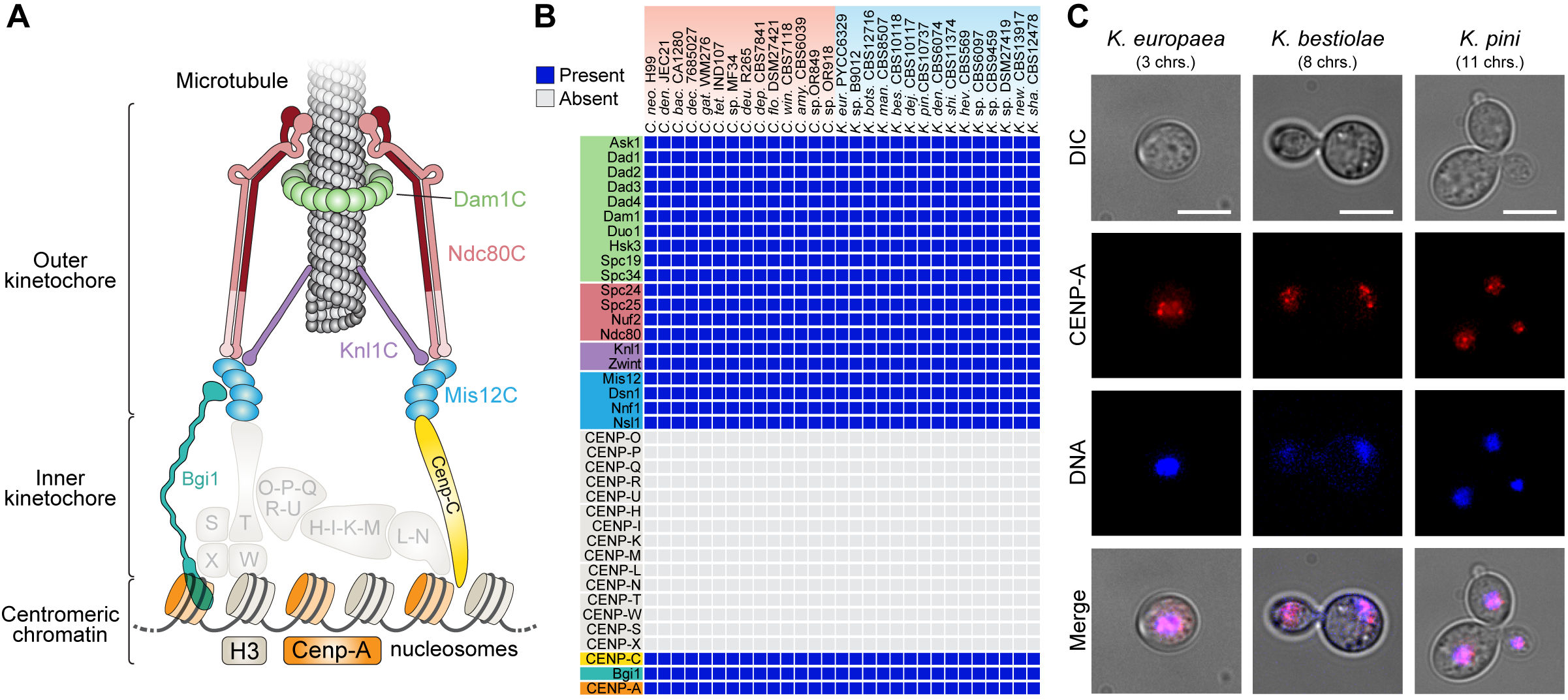

Supplement: S7 Fig — (A) Kinetochore ensemble schematic with color-coded protein complexes or faded out to indicate absence. (B) Matrix showing the presence (blue) and absence (grey) of the kinetochore components proteins depicted in panel A. (C) Live cell imaging of K. europaea, K. bestiolae, and K. pini expressing mCherry-tagged CENP-A, showing nucleus subcellular localization (scale bar, 10 μm). (TIF) [file pbio.3002682.s007.tif]

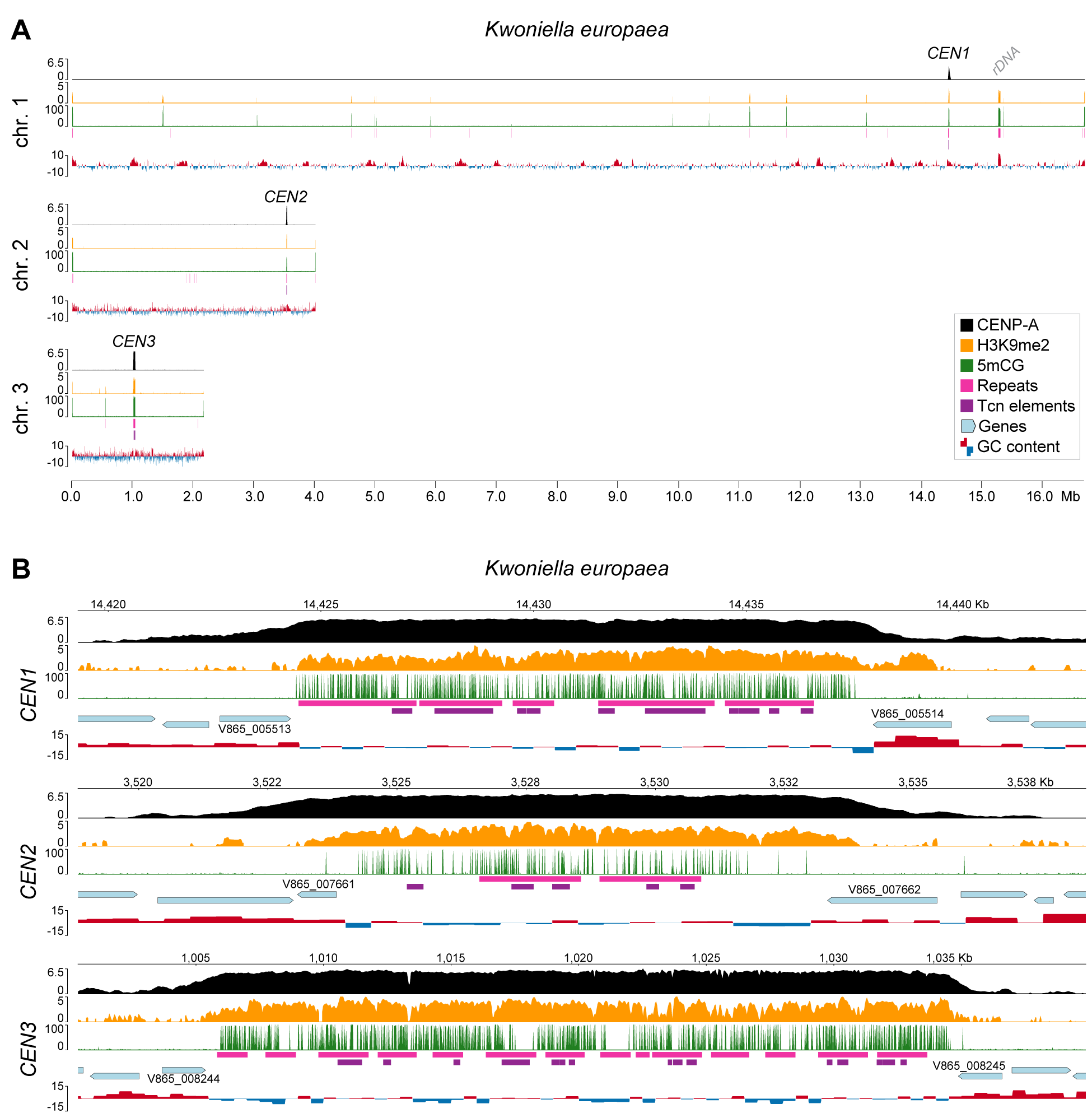

Supplement: S8 Fig — (A) Whole chromosome plots showing CENP-A (black) and H3K9me2 (orange) enrichment, CG cytosine DNA methylation (5mCG, green) derived from WGBS, repeat content (pink), TCN-like LTR elements (purple), and GC content show as deviation from the genome average (red, above; blue, below). CENP-A- and H3K9me2-enriched regions were normalized to input DNA. The data are computed in 5-kb nonoverlapping windows. (B) Zoomed-in sections show the regions spanning the centromeres and adjacent genes (light blue). Note that centromeres are enriched for CENP-A, H3K9me2, and 5mCG DNA methylation marks and contain repeat elements. (TIF) [file pbio.3002682.s008.tif]

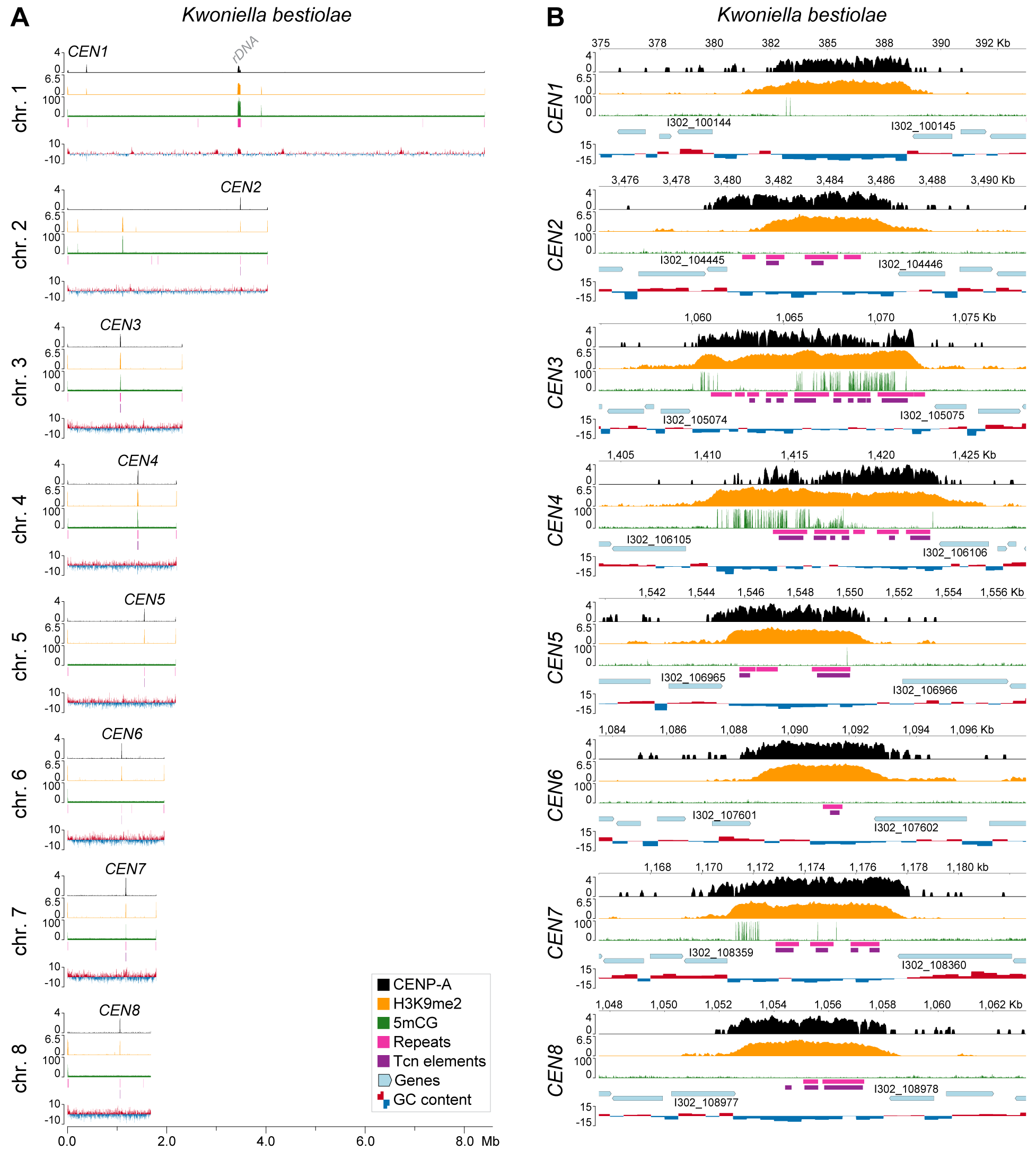

Supplement: S9 Fig — (A) Whole chromosome plots showing CENP-A (black) and H3K9me2 (orange) enrichment, CG cytosine DNA methylation (5mCG, green) derived from WGBS, repeat content (pink), TCN-like LTR elements (purple) and GC content show as deviation from the genome average (red, above; blue, below). CENP-A- and H3K9me2-enriched regions were normalized to input DNA. (B) Zoomed-in sections show the regions spanning the centromeres and adjacent genes (light blue). Note that centromeres are enriched for both CENP-A and H3K9me2 marks but 5mCG enrichment was only observed in a subset of centromeres, and even in these cases, it was localized to specific regions instead of the whole-centromere. The data are computed in 5-kb nonoverlapping windows. (TIF) [file pbio.3002682.s009.tif]

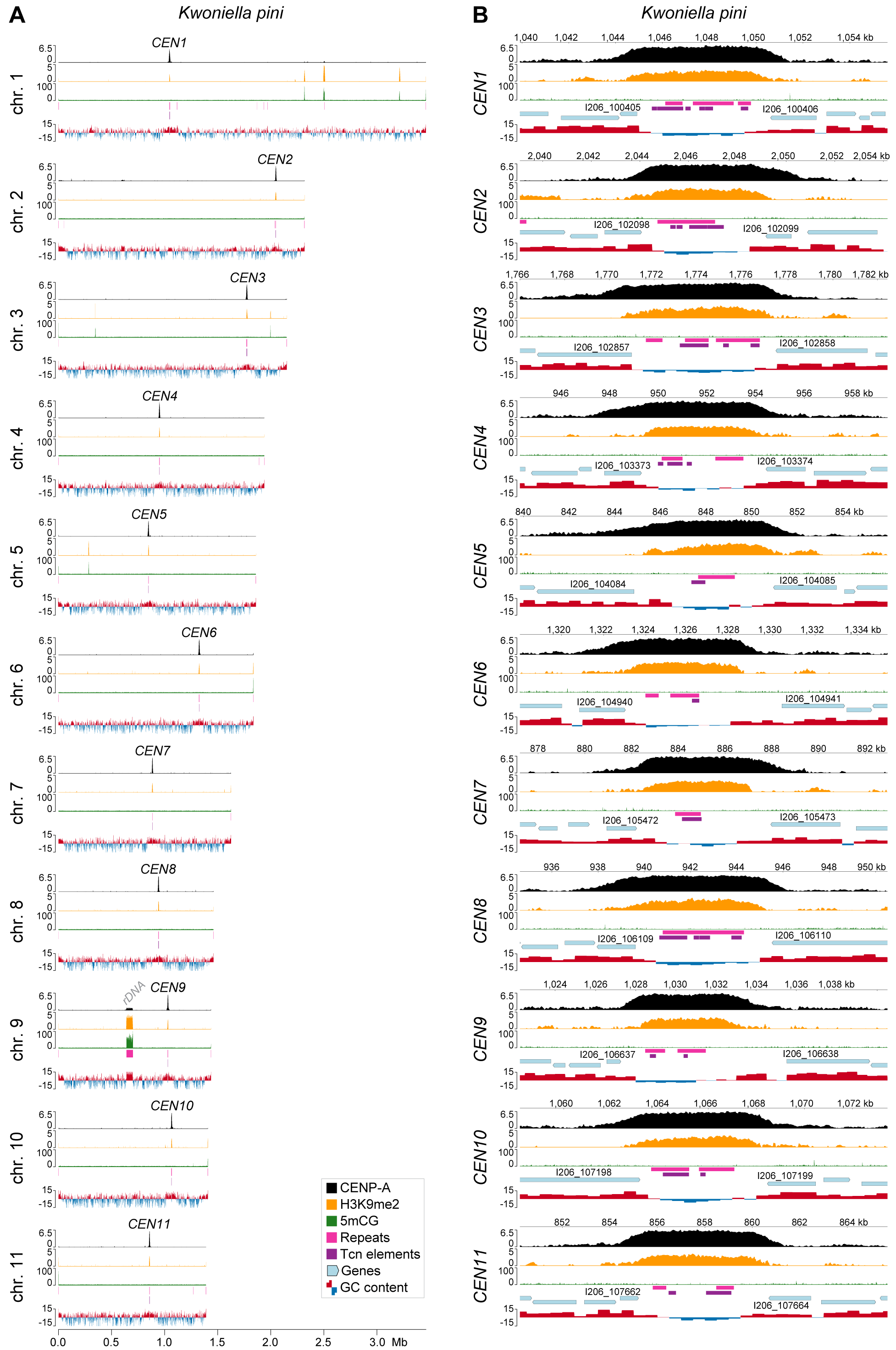

Supplement: S10 Fig — (A) Whole chromosome plots showing CENP-A (black) and H3K9me2 (orange) enrichment, CG cytosine DNA methylation (5mCG, green) derived from WGBS, repeat content (pink), TCN-like LTR elements (purple) and GC content show as deviation from the genome average (red, above; blue, below). CENP-A- and H3K9me2-enriched regions were normalized to input DNA. (B) Zoomed-in sections show the regions spanning the centromeres and adjacent genes (light blue). Note that centromeres are enriched for both CENP-A and H3K9me2 marks but are completely devoid of 5mCG DNA methylation despite the presence of this heterochromatic mark in other genomic regions. The data are computed in 5-kb nonoverlapping windows. (TIF) [file pbio.3002682.s010.tif]

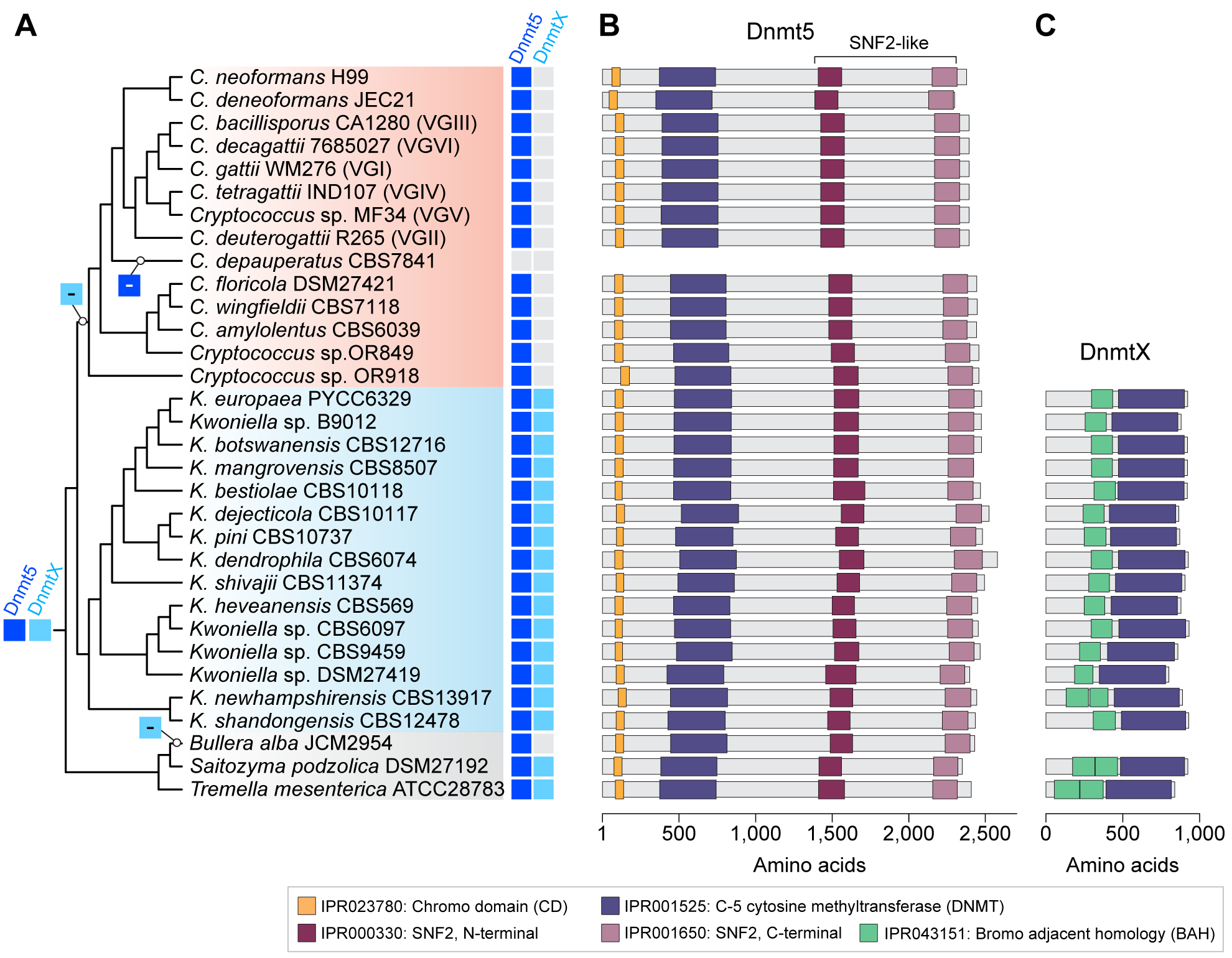

Supplement: S11 Fig — (A) Species tree topology indicating the presence/absence of 2 previously characterized DNA methyltransferases: Dnmt5 (encoded by DMT5 gene) is a maintenance-type DNA methyltransferase and DnmtX (encoded by DMTX gene) is a de novo methylase. Phylogenetic and BLAST analysis confirmed initial reports that the ancestral species of the 2 clades likely had both genes, but DMTX was lost in the Cryptococcus common ancestor, including in the early-branching Cryptococcus sp. OR918 lineage analyzed in this study. The DMTX gene is also absent in Bullera alba, indicating additional losses are expected to have occurred within the Tremellomycetes. The only instance of DMT5 loss in our dataset is observed in C. depauperatus, and our Nanopore data confirm absence of 5mC methylation in this species. (B) Similar structure of Dnmt5 across species, characterized by an N-terminal chromodomain (CD) followed by a cytosine methyltransferase catalytic domain (DNMT), and a domain related to those of SNF2-type ATPases (C). The DnmtX protein is shorter and contains a bromo-associated homology (BAH) domain and a DNMT catalytic domain. (TIF) [file pbio.3002682.s011.tif]

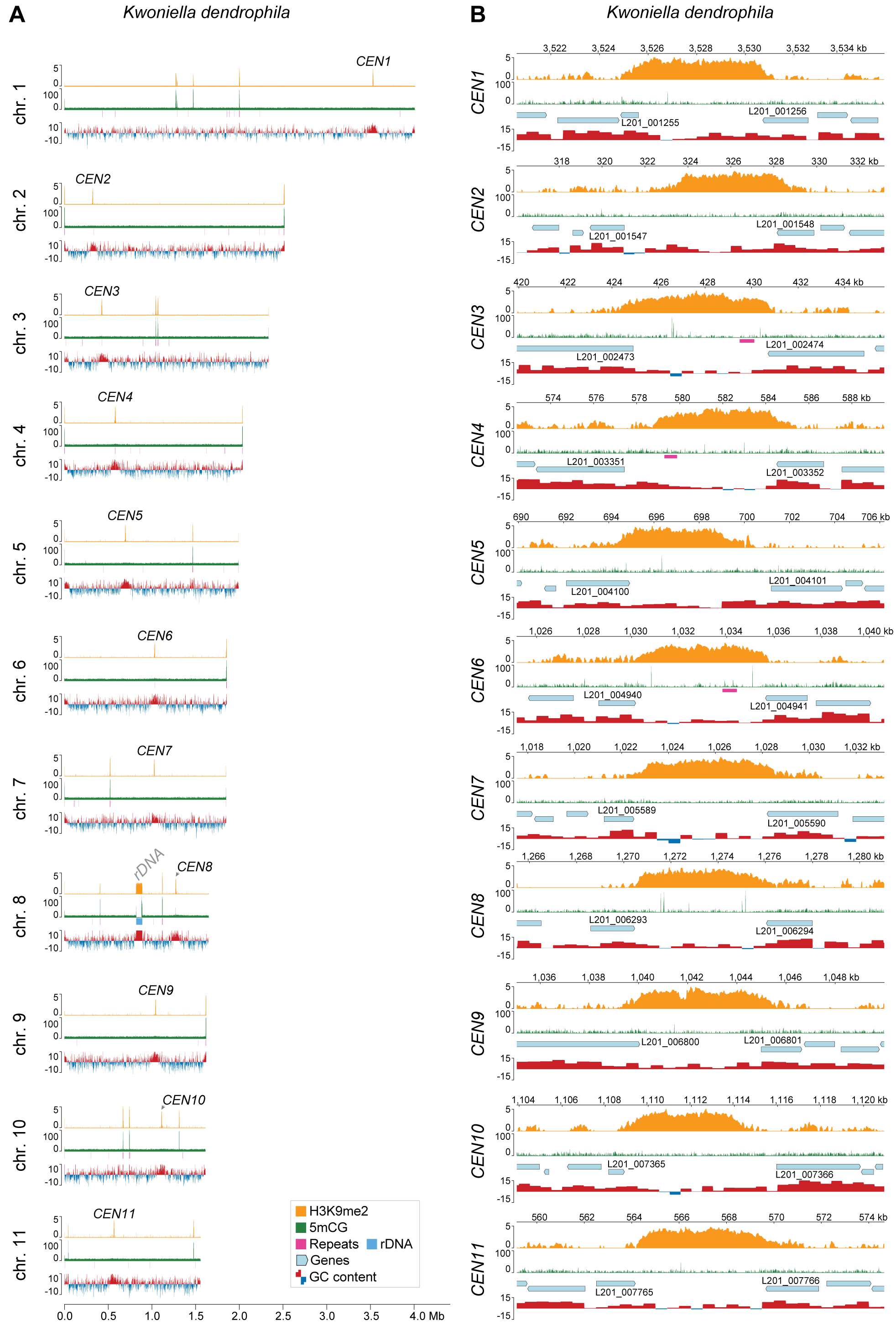

Supplement: S12 Fig — (A) Whole chromosome plots displaying H3K9me2 enrichment (orange), CG cytosine DNA methylation (5mCG, green) from WGBS, repeat content (pink), and GC content show as deviation from the genome average (red, above; blue, below). H3K9me2-enriched regions were normalized to input DNA. (B) Close-ups of predicted centromeres and adjacent genes (light blue) highlights H3K9me2 enrichment but absence of 5mCG methylation and transposable elements. The data are computed in 5-kb nonoverlapping windows. (TIF) [file pbio.3002682.s012.tif]

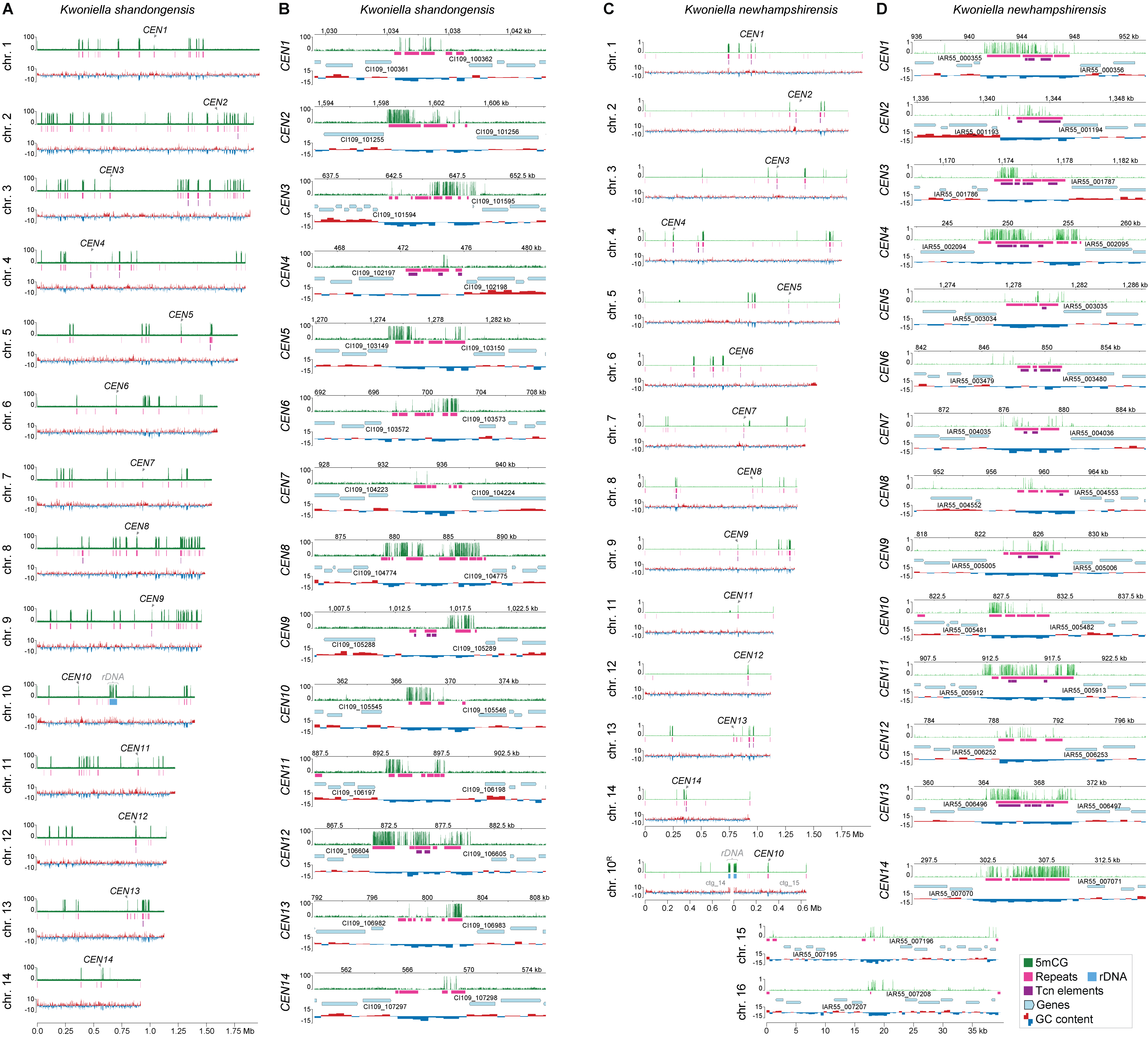

Supplement: S13 Fig — (A and C) Whole chromosome plots displaying CG cytosine DNA methylation (5mCG, green) from WGBS (K. shandongensis) or ONT data (K. newhampshirensis), alongside repeat content (pink), and GC content show as deviation from the genome average (red, above; blue, below). (B and D) Close-ups of predicted centromeres and adjacent genes (light blue) highlight diverse 5mCG methylation patterns. While most centromeres encompass unclassified repeats, only a few contain LTR retrotransposons (purple). In K. newhampshirensis, chr. 10 corresponds to assembled contigs 14 and 15, broken at the rDNA array, and chrs. 15 and 16 are mini-chromosomes with yet-to-be-determined centromere positions; however, these might correspond to the regions high in 5mCG and low in GC content. The data are computed in 5-kb nonoverlapping windows. (TIF) [file pbio.3002682.s013.tif]

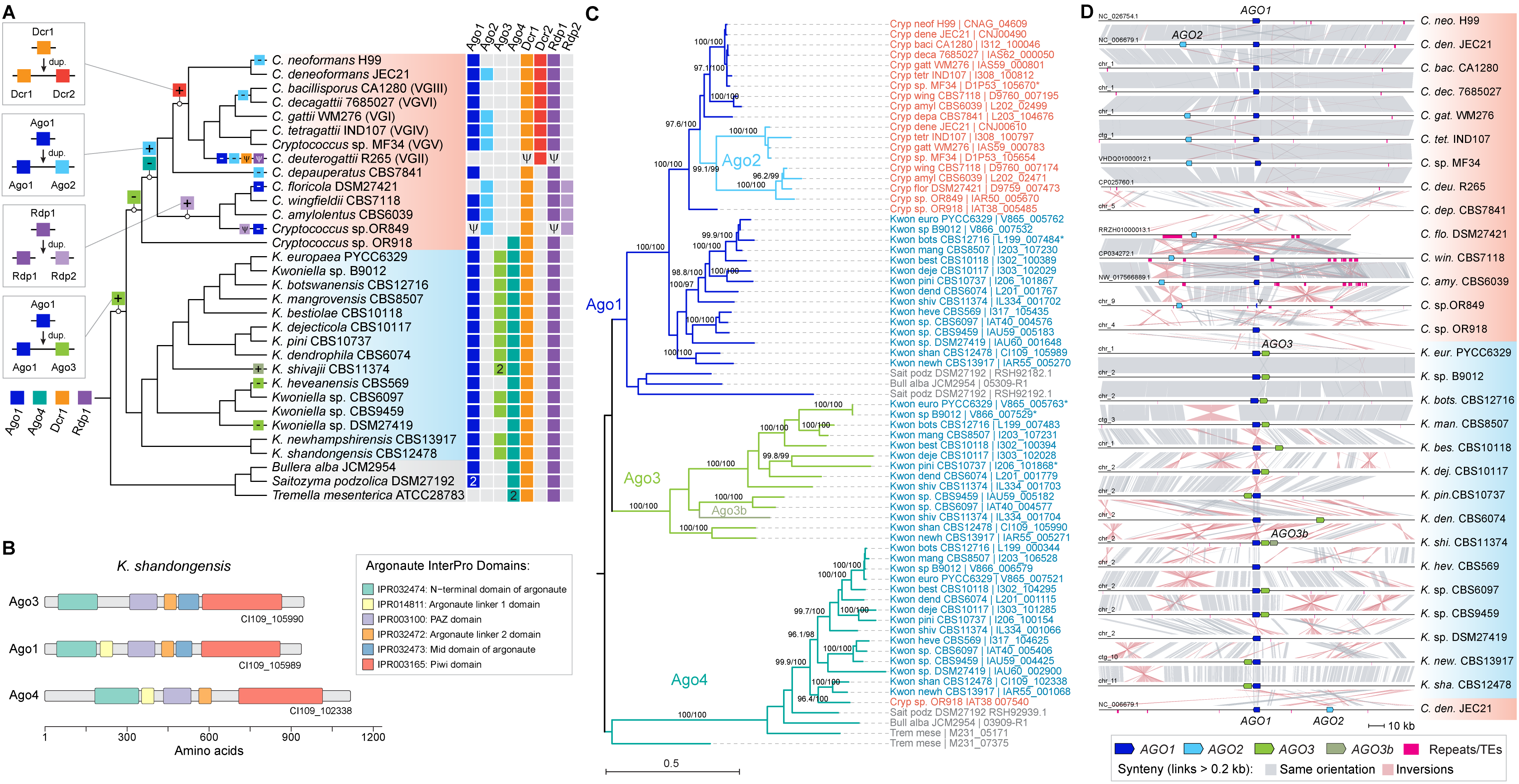

Supplement: S14 Fig — (A) Species tree topology indicating the presence/absence of the core RNAi components (Argonaute, Dicer, and RNA-dependent RNA polymerase) across species as well as the inferred pattern of gene gain (via duplication) and loss during evolution. The common ancestor of the 2 groups was an RNAi-proficient organism, likely expressing 2 Argonaute proteins (Ago1 and Ago4), 1 Dicer (Dcr1), and 1 RNA-dependent RNA polymerase (Rdp1). Psi symbols indicate pseudogenization events and numbers indicate additional species-specific copies. (B) Protein domain organization of the 3 Argonaute proteins found in K. shandongensis, depicted here as a representative. (C) ML phylogeny of the different Argonaute proteins. The tree was constructed with IQ-TREE2 (model LG+F+R5) and rooted at the midpoint. Internal branch support was assessed by 10,000 replicates of the Shimodaira–Hasegawa approximate likelihood ratio test (SH-aLRT) and ultrafast bootstrap (UFboot). Branch lengths are given in number of substitutions per site. The tree file is provided at https://doi.org/10.5281/zenodo.11199354. (D) Genomic region containing the AGO1, AGO2, and AGO3 genes across species. For simplicity, all other genes were omitted. Within the clade comprising C. floricola, C. wingfieldii, C. amylolentus, and Cryptococcus sp. OR849, a high prevalence of repeat elements is observed in this region, which may have contributed to the loss of AGO1 in C. floricola and its pseudogenization in Cryptococcus sp. OR849. (TIF) [file pbio.3002682.s014.tif]

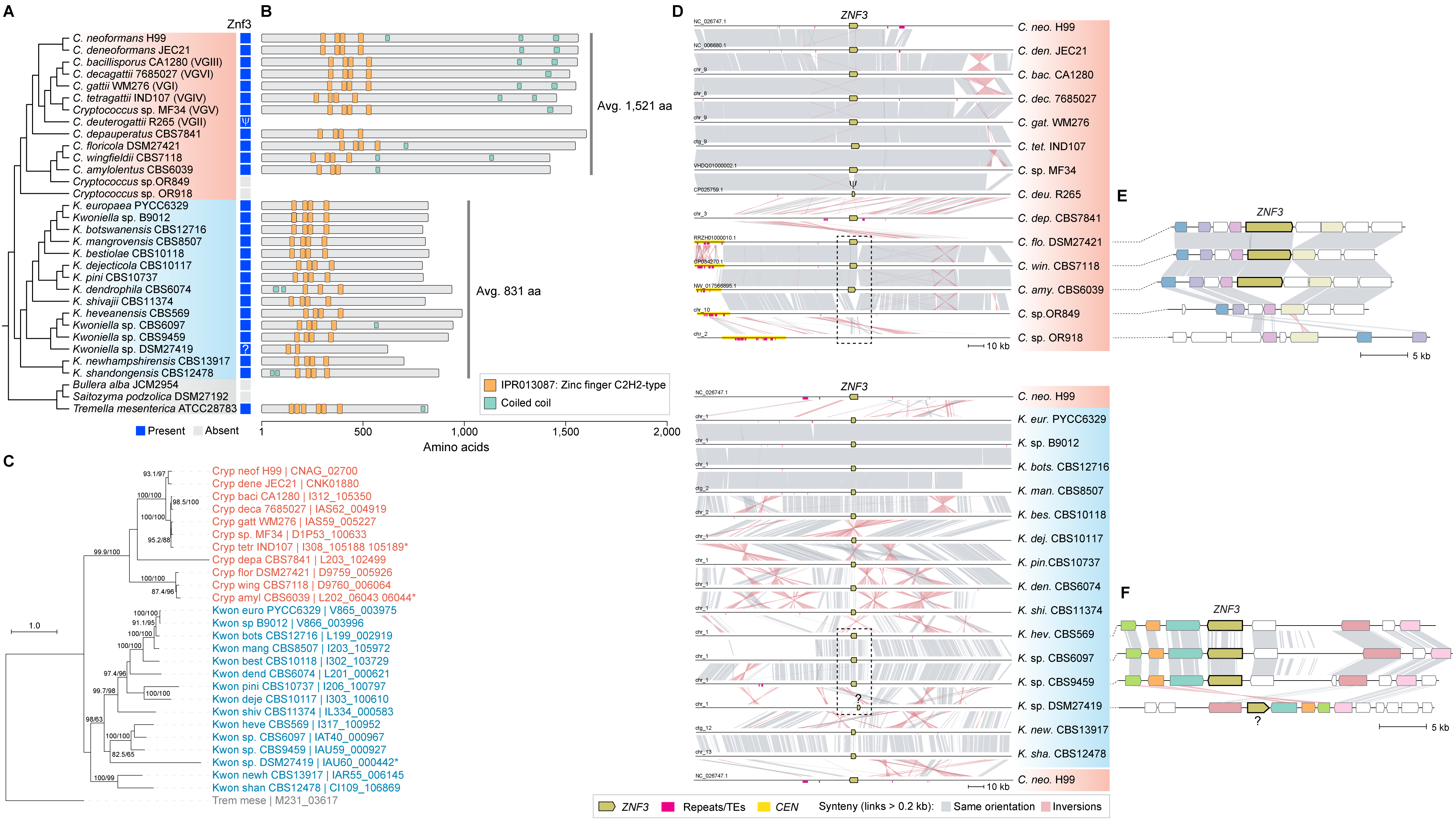

Supplement: S15 Fig — (A) Species tree topology indicating the presence/absence of Znf3 across species. (B) Protein domain organization of Znf3 proteins. Most of the proteins exhibit 4 C2H2 zinc finger domains and a few conserved coiled coil regions, often involved in protein–protein interactions. (C) ML phylogeny of the different Znf3 proteins. The tree was constructed with IQ-TREE2 (model JTT+F+I+G4) and rooted with T. mesenterica. Internal branch support was assessed by 10,000 replicates of the Shimodaira–Hasegawa approximate likelihood ratio test (SH-aLRT) and ultrafast bootstrap (UFboot). Branch lengths are given in number of substitutions per site. The tree file is provided at https://doi.org/10.5281/zenodo.11199354. (D) Genomic region encompassing the ZNF3 gene across species. For simplicity, all other genes were omitted. The genomic region containing the ZNF3 in C. neoformans H99 was plotted 3 times for comparison. (E) Detailed view depicting newly identified losses of ZNF3 in Cryptococcus sp. OR849 and Cryptococcus sp. OR918. (F) Detailed view depicting a smaller ZNF3 gene in Kwoniella sp. DSM27419. Colored genes in panels E and F denote those consistently present across all species within the compared region. (TIF) [file pbio.3002682.s015.tif]

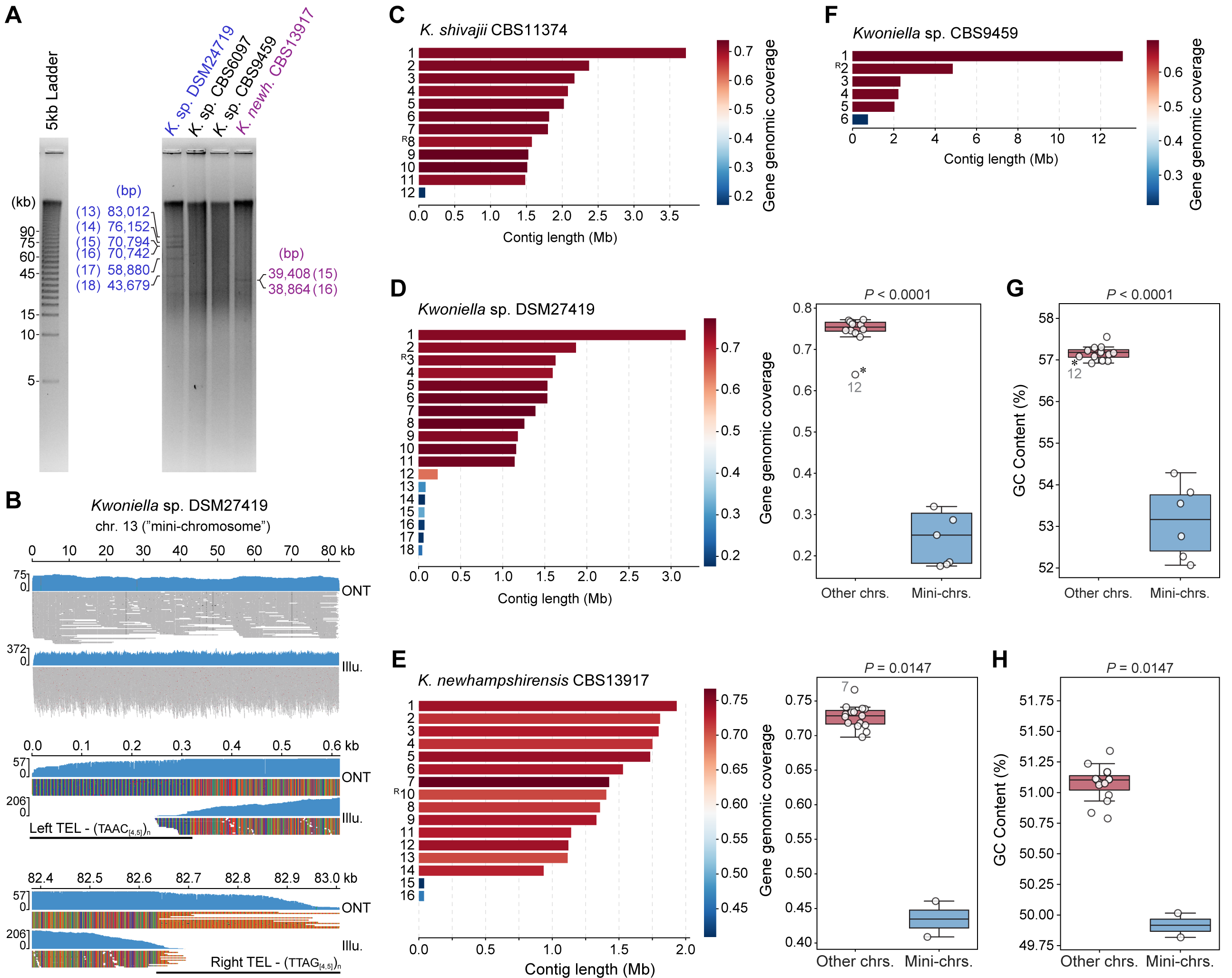

Supplement: S16 Fig — (A) Validation of “mini-chromosomes” (less than 100 kb) via PFGE in Kwoniella sp. DSM27419 and K. newhampshirensis CBS13917, and their absence in Kwoniella sp. CBS6097 and Kwoniella sp. CBS9459. Contig sizes and respective chromosome numbers are shown next to the gel lane. (B) Example of a mini-chromosome assembly validation, evidenced by uniform coverage mapping (blue areas) of both short (Illumina) and long (ONT) reads spanning the full chromosome. The panels below show close-up views of the left and right ends of the mini-chromosome emphasizing the presence of telomeric repeats. (C-F) Plots displaying individual chromosome sizes, color-coded based on gene genomic coverage (defined as the ratio of total gene length on a contig to the total length of that contig). In panels D and E, the adjacent boxplots illustrate statistically significant difference (Mann–Whitney U test) in gene genomic coverage between mini-chromosomes and other chromosomes. (G, H) Boxplots showing a notable difference (Mann–Whitney U test) in GC content between mini-chromosomes and other chromosomes in Kwoniella sp. DSM27419 and K. newhampshirensis CBS13917, respectively. While chr. 12 of Kwoniella sp. DSM27419 was categorized as a non-mini-chromosome in these analyses (larger than 100 kb and, thus, not visible in the gel in panel A), it exhibits intermediate gene genomic coverage. Chromosomes containing rDNA are indicated by an R and their size is likely underestimated. The data underlying this Figure can be found in S1 Appendix and at https://doi.org/10.5281/zenodo.11199354. (TIF) [file pbio.3002682.s016.tif]

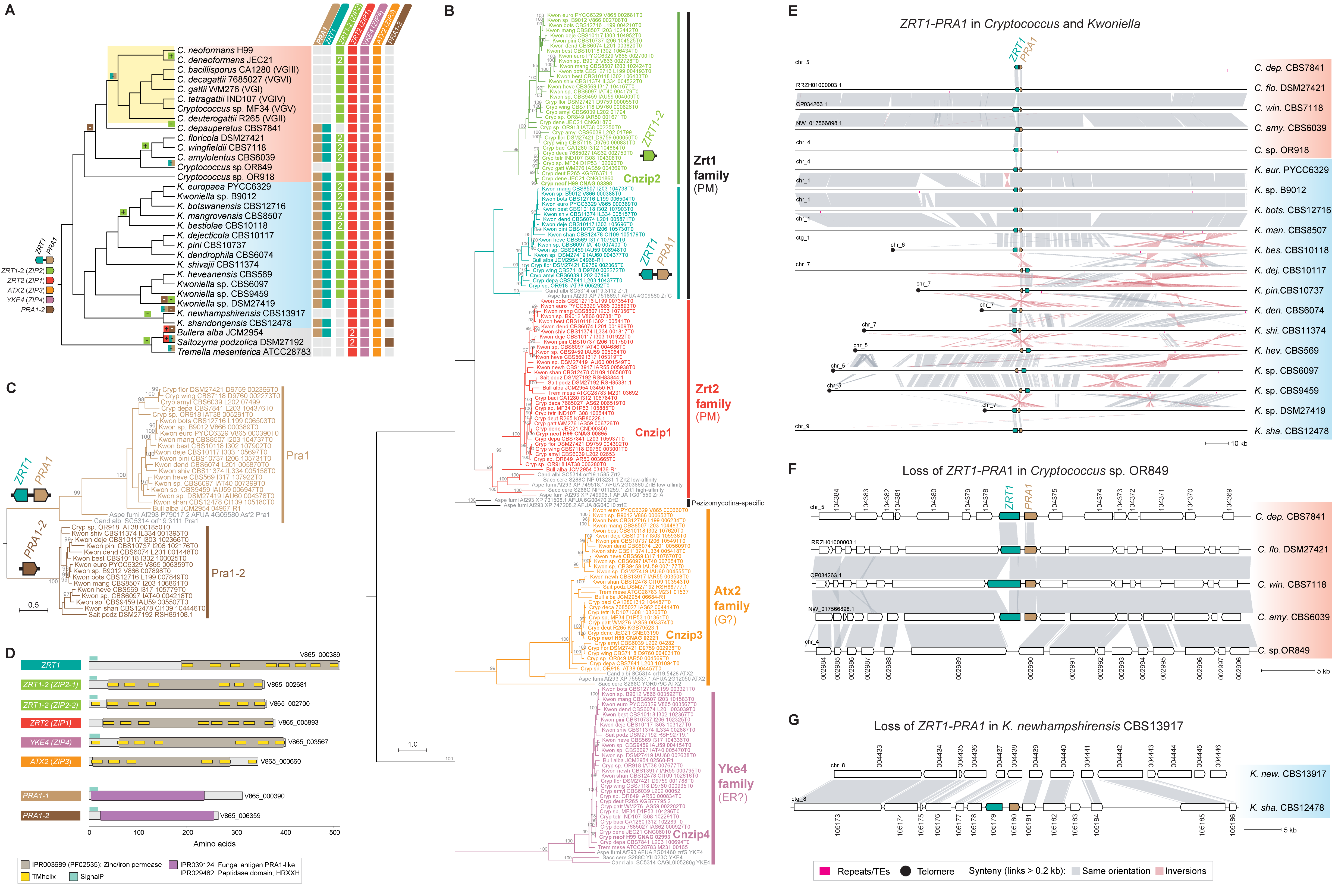

Supplement: S17 Fig — (A) Species tree topology illustrating the evolutionary trajectory of zinc transporter families and the Pra1 zincophore in Cryptococcus and Kwoniella. Gene presence in extant species is indicated in color, while absence is shown in grey. (B) ML phylogeny of major zinc-related transporters in our dataset. The tree shows a distinct separation into 5 groups. Orthologs from Saccharomyces cerevisiae, Candida albicans, and Aspergillus fumigatus, previously characterized (shown in grey), are included for comparative purposes and to aid in functional prediction. Genes included in the Zrt1 and Zrt2 families are predicted to transport zinc and localize to the plasma membrane (PM); those included in the Atx2 family may have more affinity to manganese transport and localize to Golgi (G); and those within the Yke4 family may function as bidirectional zinc transporters located in the endoplasmic reticulum (ER). The Zrt1 family encompasses 2 different sets of proteins: Zrt1 and Zip2. The ZRT1 gene is always clustered with PRA1, whereas ZIP2 is found elsewhere in the genome. Note that the ZRT1-PRA1 gene cluster was lost in all of the Cryptococcus pathogenic species. (C) ML phylogeny of Pra1 with sequences identified across Cryptococcus and Kwoniella. A diverged copy of PRA1 (labelled as PRA1-2) is present in the early derived Cryptococcus sp. OR918 as well as in most Kwoniella species. Midpoint rooted phylogenetic trees in panels B and C, with branch lengths representing number of substitutions per site, were constructed with IQ-TREE2 (using models LG+R6 and WAG+G4, respectively), and with internal branch support assessed by 10,000 replicates of Shimodaira–Hasegawa approximate likelihood ratio test (SH-aLRT) and ultrafast bootstrap (UFboot). The tree files are provided at https://doi.org/10.5281/zenodo.11199354. (D) Protein domains of zinc transporters and putative Pra1 zincophores identified in K. europaea, shown here as an example. (E) Genomic region encompassing the ZRT1-PRA1 ge [file pbio.3002682.s017.tif]

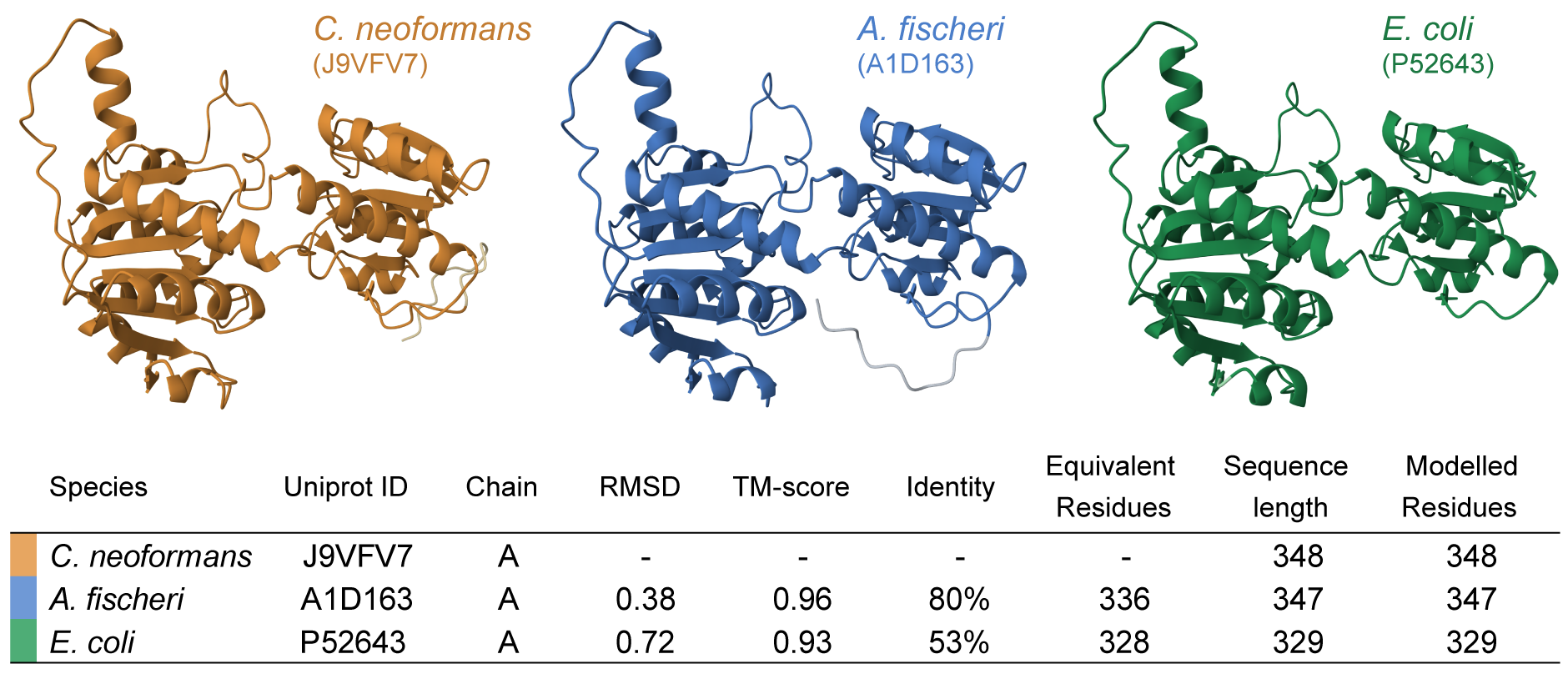

Supplement: S18 Fig — AlphaFold-predicted structures of Cryptococcus neoformans (UniProt J9VFV7), Aspergillus fischeri (UniProt A1D163), and Escherichia coli (UniProt P52643) are individually displayed at the top. Pairwise structure alignments with the C. neoformans protein were conducted with the JjFATCAT-rigid algorithm on the Protein Data Bank website (https://www.rcsb.org/alignment). The table below presents the resulting root mean square deviation (RMSD) and template modeling (TM) scores, among other metrics. The comparison reveals high structural similarity across these proteins, evidenced by low RMSD and high TM scores. The notably lower RMSD score between the 2 fungal proteins, aligns with the proposed hypothesis of a horizontal gene transfer event from an Aspergilli donor lineage to pathogenic Cryptococcus species. (TIF) [file pbio.3002682.s018.tif]
