# Supplementary material for: Comparative genomics of the closely related fungal genera Cryptococcus and Kwoniella reveals karyotype dynamics and suggests evolutionary mechanisms of pathogenesis
Source: PLoS Biol. 2024 Jun 6;22(6):e3002682. doi: 10.1371/journal.pbio.3002682 (PMC11185503; doi:10.1371/journal.pbio.3002682)
Supplement: S1 Raw Images — (PDF) [file pbio.3002682.s028.pdf]

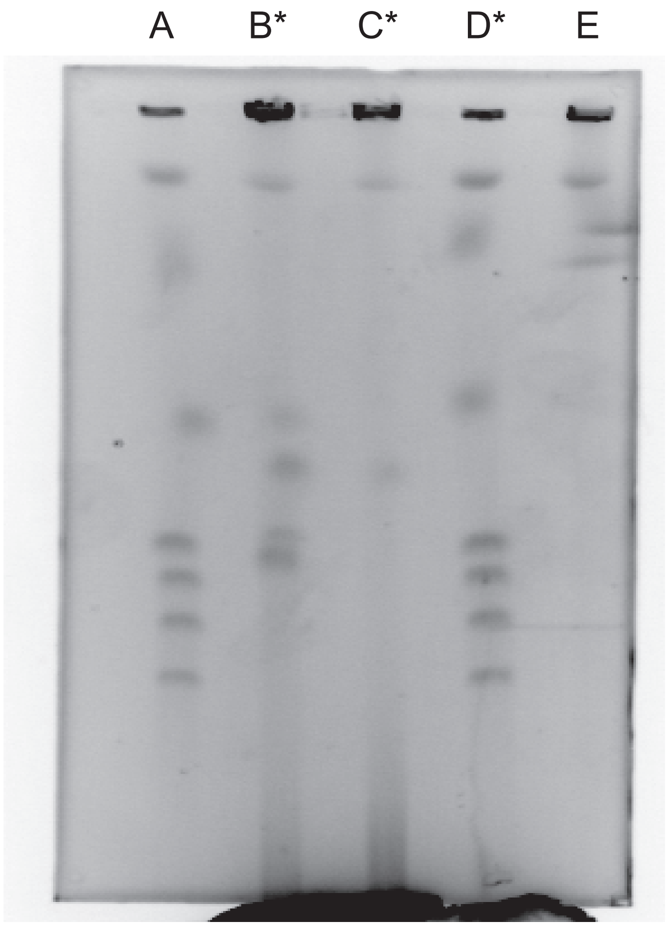

A - *Hansenula wingei* (marker)
B - *Kwoniella bestiolae* CBS10118
C - *Kwoniella europaea* PYCC6329
D - *Hansenula wingei* (marker)
E - *Schizosaccharomyces pombe* (marker)

Lanes depicted in **S2A Fig** are indicated by asterisks on the original gel

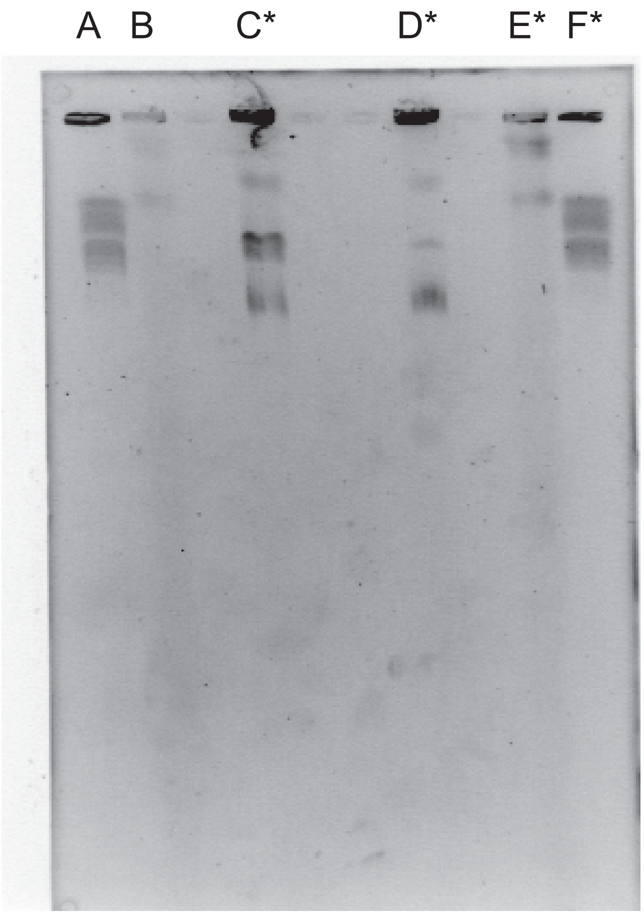

A - *Hansenula wingei* (marker)
B - *Schizosaccharomyces pombe* (marker)
C - *Kwoniella bestiolae* CBS10118
D - *Kwoniella europaea* PYCC6329
E - *Schizosaccharomyces pombe* (marker)
F - *Hansenula wingei* (marker)

Lanes depicted in **S2B Fig** are indicated by asterisks on the original gel.

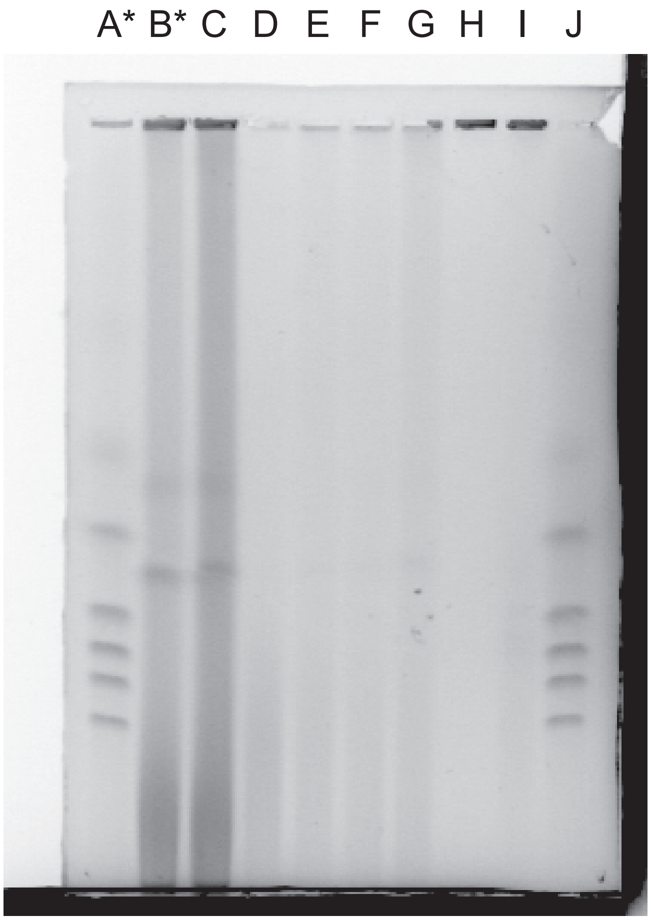

A - *Hansenula wingei* (marker)
B - *Kwoniella mangrovensis* CBS8507 (rep1)
C - *Kwoniella mangrovensis* CBS8507 (rep2)
D - *Kwoniella* sp. B9012 (rep1)
E - *Kwoniella* sp. B9012 (rep1)
F - *Kwoniella botswanensis* CBS12716 (rep1)
G - *Kwoniella botswanensis* CBS12716 (rep2)
H - *Kwoniella europaea* PYCC6329
I - *Kwoniella bestiolae* CBS10118
J - *Hansenula wingei* (marker)

Lanes depicted in **S2C Fig** are indicated by asterisks on the original gel.

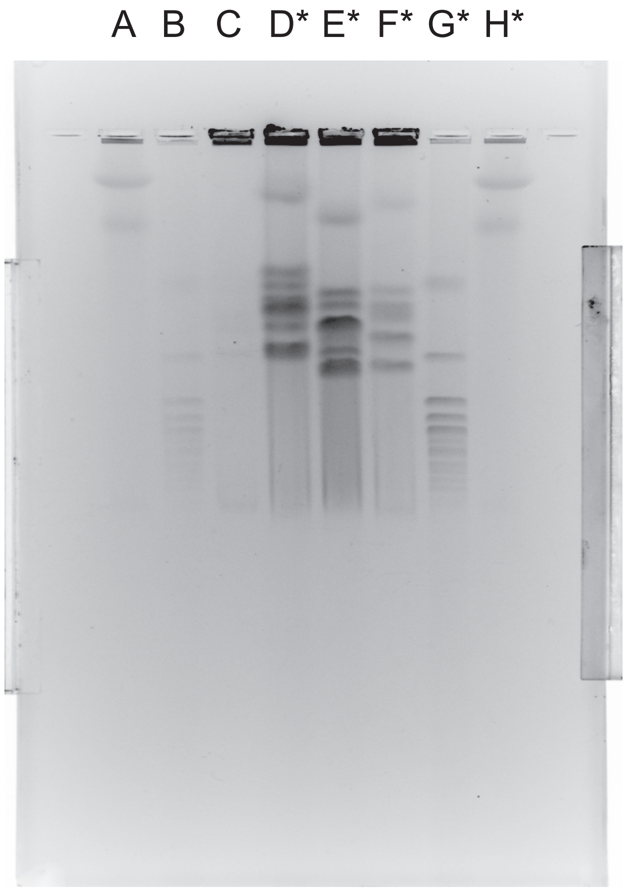

A - *Schizosaccharomyces pombe* (marker)
B - *Saccharomyces cerevisiae* (marker)
C - *Kwoniella dendrophila* CBS6074
D - *Kwoniella dejecticola* CBS10117
E - *Kwoniella pini* CBS 10737
F - *Kwoniella shivajii* CBS11374
G - *Saccharomyces cerevisiae* (marker)
H - *Schizosaccharomyces pombe* (marker)

Lanes depicted in **S2D Fig** are indicated by asterisks on the original gel.

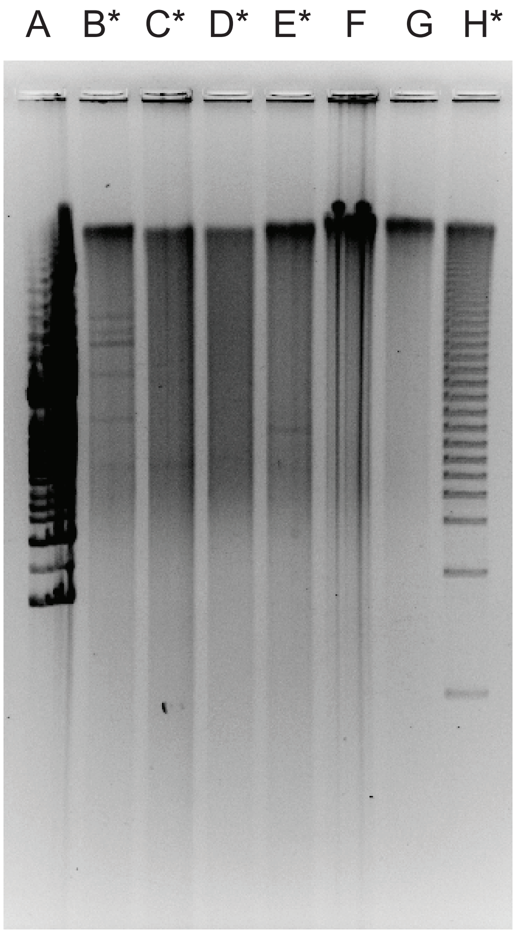

A - 8-48 kb ladder (marker)
B - *Kwoniella* sp. DSM27419
C - *Kwoniella* sp. CBS6097
D - *Kwoniella* sp. CBS9459
E - *Kwoniella newhampshirensis* CBS13917
F - *Cryptococcus depauperatus* CBS7841
G - *Cryptococcus depauperatus* CBS7855
H - 5 kb ladder (marker)

Lanes depicted in **S16A Fig** are indicated by asterisks on the original gel.
